# Rapid Preparation of MWCNTs/Epoxy Resin Nanocomposites by Photoinduced Frontal Polymerization

**DOI:** 10.3390/ma13245838

**Published:** 2020-12-21

**Authors:** Guofeng Hu, Wanli Fu, Yumin Ma, Jianping Zhou, Hongbo Liang, Xinmei Kang, Xiaolin Qi

**Affiliations:** 1 School of Material Science and Engineering, Nanchang Hangkong University, Nanchang 330063, China; 1801085204012@stu.nchu.edu.cn (G.H.); 70965@nchu.edu.cn (W.F.); maym@hollowlite.com (Y.M.); lhongbo@nchu.edu.cn (H.L.); 2 State-Owned Assets Management Division, Nanchang Hangkong University, Nanchang 330063, China; 3 Jiangxi Provincial Engineering Research Center for Surface Technology of Aeronautical Materials, Nanchang Hangkong University, Nanchang 330063, China; 4 Aviation Key Laboratory of Science and Technology on Life-Support Technology, Xiangyang 441000, China; kangkang198306@163.com (X.K.); ajian421@126.com (X.Q.)

**Keywords:** epoxy resin, carbon nanotubes, nanocomposite, frontal polymerization, properties

## Abstract

Due to their excellent mechanical and thermal properties and medium resistance, epoxy/carbon nanotubes and nanocomposites have been widely used in many fields. However, the conventional thermosetting process is not only time- and energy-consuming, but also causes the agglomeration of nanofillers, which leads to unsatisfactory properties of the obtained composites. In this study, multi-walled carbon nanotubes (MWCNTs)/epoxy nanocomposites were prepared using UV photoinduced frontal polymerization (PIFP) in a rapid fashion. The addition of MWCNTs modified by a surface carboxylation reaction was found to enhance the impact strength and heat resistance of the epoxy matrix effectively. The experimental results indicate that with 0.4 wt % loading of modified MWCNTs, increases of 462.23% in the impact strength and 57.3 °C in the glass transition temperature *T*_g_ were achieved. A high-performance nanocomposite was prepared in only a few minutes using the PIFP approach. Considering its fast, energy-saving, and environmentally friendly production, the PIFP approach displays considerable potential in the field of the fast preparation, repair, and deep curing of nanocomposites and coatings.

## 1. Introduction

Owing to their great aspect ratio, excellent mechanical properties, electrical conductivity, and chemical/thermal stability, carbon nanotubes (CNTs) have been extensively studied by researchers in the field of materials science [1,2,3,4,5]. However, due to their extremely high surface energy, CNTs are difficult to evenly disperse during the preparation of CNT-doped composite materials, which has greatly limited their wide application in the field of composites [6]. Therefore, the surface modification of CNTs is particularly necessary in the preparation of CNT-doped composites [7,8,9,10]. Currently, two modifying methods have been developed to improve the dispersion of CNTs: noncovalent and covalent modification. The former mainly includes ball milling, mechanical stirring, and ultrasonication [11,12,13]; the latter mainly involves the introduction of reactive groups, such as carboxyl, hydroxyl, and amino groups, onto the surface of CNTs by oxidizing and etching under the action of H_2_SO_4_ and HNO_3_ or other strong oxidants [14,15].

Epoxy resin (EP) is one of the most widely used thermosetting resins due to its excellent mechanical and bonding properties, chemical resistance, and processing simplicity [16,17,18,19]. However, its cured products usually have a high crosslinking density, making them vulnerable to external force impacts. To overcome the relatively poor impact resistance and environmental stress-cracking resistance of epoxy condensates, the addition of inorganic nanoparticles, glass fibers, graphene, and CNTs to epoxy resin has been extensively explored [20,21,22,23,24,25]. Particularly, using modified CNTs as fillers to prepare CNTs/epoxy nanocomposites has been reported [26,27]. For example, Akram et al. constructed epoxy nanocomposites with polyacrylonitrile/multi-walled CNTs and a polyacrylonitrile/carbon nanofiber aerogels scaffold [28].

Frontal polymerization (FP) is a new type of polymerization that can transform monomers into polymers quickly through an exothermic reaction [29,30]. Compared to the traditional polymerization reaction, FP exhibits various advantages: it has a high reaction rate and a short reaction time; it consumes a low amount of energy; and it is environmentally friendly [31]. Frontal polymerization is classified into thermal frontal polymerization (TFP), photo frontal polymerization (PFP), and isothermal frontal polymerization (IFP) [32,33]. There are now many studies on TFP and PFP [30,34,35,36,37,38]. For instance, Sangermano et al. successfully prepared a glass-fiber-reinforced epoxy resin composite through the PFP approach. Zhu et al. successfully cured tripropylene glycol diacrylate (TPGDA) through a UV-triggered frontal polymerization [39].

In this study, nanocomposites of epoxy and multi-walled carbon nanotubes (MWCNTs) were rapidly prepared via photoinduced frontal polymerization. The MWCNTs were first subjected to surface medication through a carboxylation reaction. The influence of adding the modified MWNCTs to the frontal polymerization process and the properties of the obtained nanocomposites were then further investigated in detail.

## 2. Materials and Methods

### 2.1. Materials

The epoxy resin used in this study was 3,4-epoxycyclo-hexyl formate 3′,4′-epoxycyclohexyl methyl ester (EP-211), purchased from Tianjin Synthetic Material Research Institute Co., Ltd., Tianjin, China. The carbon nanotubes used were multi-walled carbon nanotubes (diameter < 8 nm, purity > 98%), purchased from Chengdu Organic Chemicals Co., Ltd., Chinese Academy of Science, Chengdu, China. P-aminobenzoic acid (PABA, C_7_H_7_NO_2_) (McLean Biochemistry Co., Ltd., Shanghai, China) was used to modify the surface of the MWCNTs. Isopentyl nitrite (C_5_H_11_NO_2_, McLean Biochemistry Co., Ltd., Shanghai, China) was used as the catalyst for the carboxylation modification reaction. The boron trifluoride-amine complex (Beijing Huineng Rubber Chemical Co., Ltd., Beijing, China) was used as the thermal initiator (TI), and a mixture of hexafluoroantimonate sulfonium onium salts (Changzhou Qiangli Electronic Materials Co., Ltd., Changzhou, China) was used as the photoinitiator (PI). N,N-dimethylformamide (DMF) was purchased from Xilong Science Co., Ltd., Shantou, China. All materials were used as received without further purification.

### 2.2. Surface Carboxylation Modification of MWCNTs

To enhance the compatibility of the MWCNTs with the epoxy matrix, a carboxylation reaction was conducted. Firstly, MWCNTs were dispersed in distilled water at room temperature for 1 h using an ultrasonic disperser. Then, a certain proportion of isopentyl nitrite and various amounts of PABA ((MWCNTs)/(PABA) = 1:1, 1:5, 1:10, 1:15 wt/wt) were added to the flask, which was equipped with a condenser and a drying tube. The mixture was stirred at 80 °C for 18 h. Afterward, the resulting substances were washed with DMF five times and collected by filtration. Carboxylic-functionalized MWCNTs (MWCNTs-COOH) were finally obtained by drying in a vacuum oven at 50 °C for 24 h.

### 2.3. Preparation of MWCNTs/Epoxy Resin Nanocomposites Via UV-Light-Induced FP

The scheme of the experimental device is shown in Figure S1 in the Supplementary Materials. An optical-cable-directed UV-point light source (Shenzhen Blue Spectrum Rich Ltd., Shenzhen, China) was used to trigger the frontal polymerization, with its optical cable placed over the left end of a glass tank measuring 15 cm × 1.5 cm × 1.7 cm. Additionally, a K-type thermocouple connected to a digital recorder was used to monitor the temperature profile of the polymerization reaction.

The procedure was similar to that presented in [34], with slight modifications. During each experiment, the glass tank was first preheated in an oven set at 55 °C. Various amounts of modified MWCNTs (0.2–1 wt %), PI, and epoxy resin were added to a 50 mL round-bottom flask and mixed with a sonicator for 10 min. The flask was then transferred to a constant-temperature oil bath. The mixture in the flask was stirred at 55 °C with a magnetic stirrer, and a vacuum pump was used to degas the mixture until bubbles were no longer observed in the flask. Afterwards, a 2 wt % TI was added to the flask, and the mixture was continuously stirred for another 10 min. Finally, the mixture was quickly poured into the preheated glass tank, and the UV point source was immediately powered on at a setting of 10 W/cm^2^ to start the frontal polymerization process. Once the polymerization front formed (usually in dozens of seconds), the UV light was turned off, and the front continued to propagate horizontally to the right until all the monomers were transformed into cured products, and the MWCNTs/epoxy nanocomposites were obtained. During the reaction process, a THTZ408R-type infrared thermal imager (Tenghui Temperature Control Instrument Factor Ltd., Ningbo, China) was used to record the temperature profile and the moving feature of the polymerization front as a function of time.

### 2.4. Characterization and Measurements

The chemical structure of the modified MWCNTs and the nanocomposite products was characterized using a VERTEX70-type Fourier transform infrared spectroscopy (FT-IR, BRUKER Co., Ltd., Woodlands, Germany) and a Kratos AXIS X-ray photoelectron spectroscopy (XPS, KRATOS Co., Ltd., Kyoto, Japan). FT-IR measurements were carried out under ATR (Attenuated Total Reflection) mode with a 700–4000 cm^−1^ scanning range, accumulating 16 scans for each spectrum at 25 °C. X-ray photoelectron spectroscopy (XPS) measurements were carried out on an Escalab 250 Xi spectrometer (Thermo Scientific, Waltham, MA, USA). A monochromatic Al-Kα radiation source (1486.74 eV) and a PHOIBOS 150 hemispherical electron analyzer (SPECS, Berlin, Germany) were used with the spectrometer. An X-ray power of 25 W, a take-off angle of 45°, and a pass energy of 20 eV were used to capture 10 scans for each sample.

The viscosity measurement was performed at 25 °C in a Rotary viscometer (NDJ-79, Shanghai Changji Geological Instrument Co., Ltd., Shanghai, China) with a diameter die of 0.5 mm.

The fracture surfaces of the nanocomposite specimens after impact test were observed using an FEI Quanta250 scanning electron microscopy (SEM, FEI Co., Ltd., Brno, Czech) with an operating voltage of 15 kV. To increase the conductivity, the sample was sprayed with gold.

Thermogravimetric analysis (TGA) was performed on a Diamond TG/DTA (Perkin Elmer, Waltham, MA, USA) thermogravimetric analysis analyzer with a temperature range of 20–800 °C. The heating rate was 10 °C /min and the flow rate of nitrogen was 20 mL/min. The difference between the residual mass after degradation of modified carbon nanotubes and that of original carbon nanotubes was regarded as the grafting amount of PABA.

Dynamic thermal mechanical (DMA) tests were performed using a TA Q800 dynamic mechanical thermal analyzer (TA Instruments, New Castle, DE, USA) with a temperature range of 25–250 °C and a frequency of 1 Hz. The heating rate was 5 K/min and the flow rate of nitrogen was 30 mL/min. The dimension of the samples used for the testing was 35 mm × 12 mm × 3.5 mm.

The impact properties of the nanocomposite specimens were measured using an RG-30 impact testing machine (Zhonghang Times Instrument Equipment Co., Ltd., Beijing, China) in accordance with ASTM D256-10. Each sample was tested three times, and the results were averaged.

## 3. Results

### 3.1. Structural Characterization

**Scheme 1:**
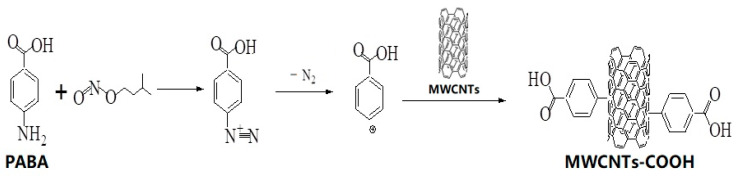
Surface modification of MWCNTs by PABA.

To enhance the dispersion and compatibility of MWCNTs in epoxy oligomers and cured products, the functionalization of MWCNTs was carried out by grafting carboxyl groups onto their surfaces. Functionalization of the initial sidewall of MWCNTs proceeded via the reaction between P-aminobenzoic acid (PABA) and the carbon nanotube surface in the presence of an oxidizing agent. As shown in Scheme 1, isoamyl nitrite first reacts with PABA to form diazonium salt of PABA. Diazonium benzoate is very unstable, and easily decomposes at a slightly higher temperature and produces a cation in its para position. The cation reacts with the double bond on the surface of the MWCNTs to graft benzoic acid group onto their surfaces.

The chemical structure of MWCNTs after being modified by PABA via carboxylation reaction was characterized by FT-IR and XPS measurements.

**Figure 1:**
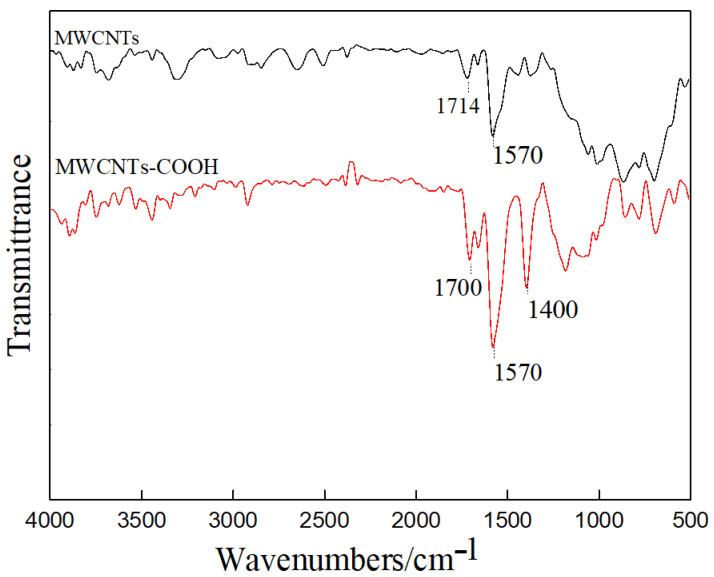
FT-IR spectra of MWCNTs before and after modification.

Figure 1 shows the FT-IR spectra of MWCNTs before and after the surface modification. Two new peaks appeared in the PABA-modified MWCNTs curve: the peak at 1700 cm^−1^ corresponded to carbonyl groups (C=O), and the peak at 1400 cm^−1^ corresponded to carboxyl groups (–COO) [25]. The presence of these two peaks indicated that PABA had been grafted successfully onto the surface of the MWCNTs.

**Figure 2:**
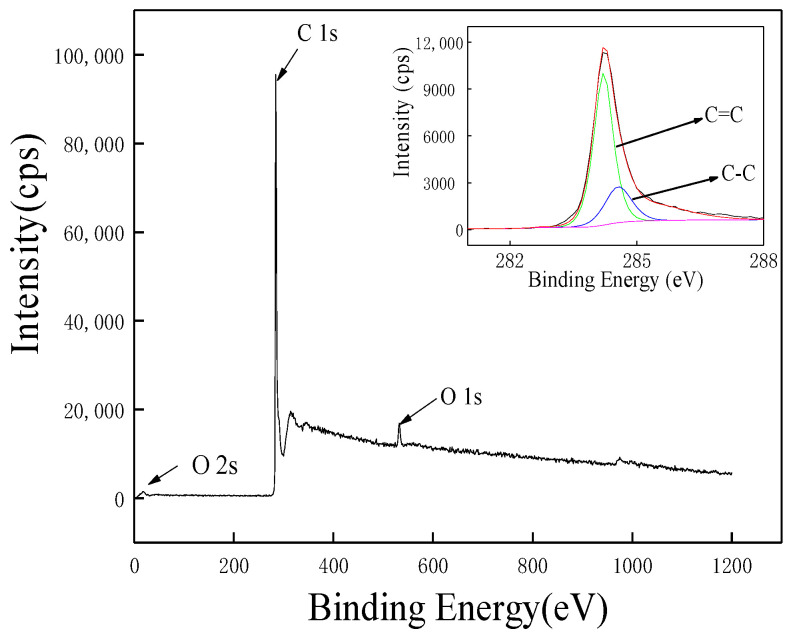
XPS spectra for unmodified MWCNTs. The inset shows the C1s spectra of prime MWCNTs.

**Figure 3:**
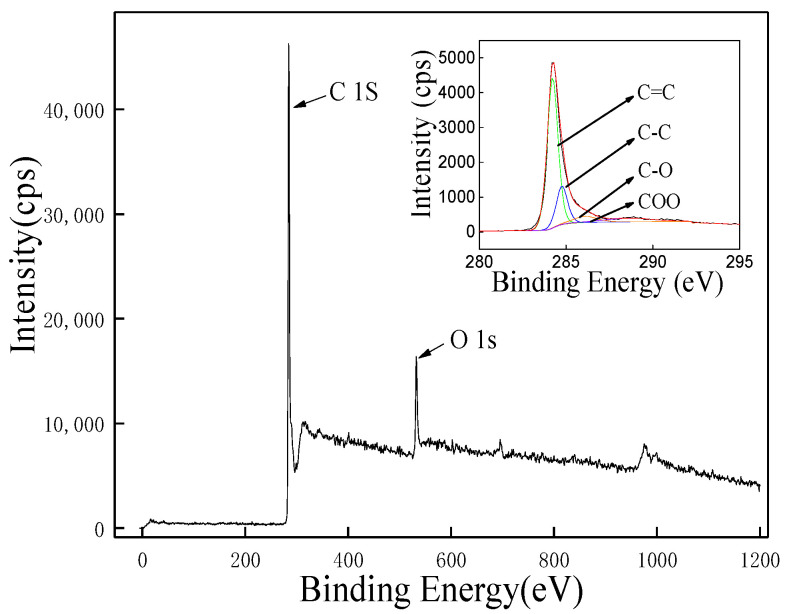
XPS spectra for PABA-modified MWCNTs. The inset shows the C1s spectra of modified MWCNTs.

XPS analysis is an efficient method to evaluate the chemical bonds grafted onto CNTs [40]. Figure 2 and Figure 3 show the XPS spectra of prime and PABA-modified MWCNTs. The inset graph in Figure 2 shows the deconvolution of the C1s spectrum of prime MWCNTs, in which the main peak at 284.6 ± 0.2 eV was assigned to the graphitic C–C bond and the 284.1 ± 0.2 eV was assigned to the C=C bond. In contrast, the C1s spectrum of the modified MWCNTs shown in the inset of Figure 3 demonstrates the existence of an –O–C=O bond (corresponding to the peak at 288.8 ± 0.2 eV) and a C–O bond (corresponding to the peak at 286.5 ± 0.2 eV), which also indicated the successful grafting of PABA onto the surface of MWCNTs.

**Figure 4:**
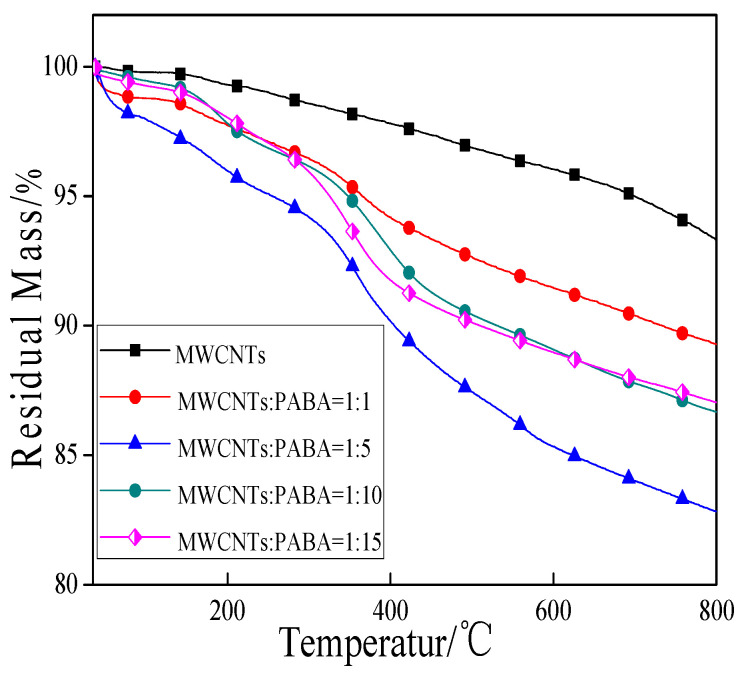
TGA curves of prime MWCNTs and PABA-modified MWCNTs.

Figure 4 shows the TGA curves of prime and modified MWCNTs. The PABA-modified MWCNTs underwent a three-step degradation. The first degradation occurred below 200 °C, which could mainly be due to the evaporation of adsorbed and bounded water. The second degradation occurred in the temperature range of 200–260 °C, which could be attributed to the degradation of PABA, which was suggested by the degradation curve of pure PABA (as shown in Figure S2). The third degradation step, in the temperature range of 260–800 °C, was mainly due to the thermal decomposition of the C–C bonds of MWCNTs. Using the thermogram of the PABA-modified MWCNTs, the optimum ratio of MWCNTs/PABA that could be used in the modification reaction was determined to be 1:5, as the residual mass of the modified MWCNTs was the minimum in this case. The corresponding surface grafting rate of PABA was calculated to be 10.6%. Furthermore, the grafting rate was found to decrease when the ratio of MWCNTs/PABA exceeded 1:5, implying that an excess of PABA input is not favorable for the surface carboxylation reaction between MWCNTs and PABA molecules.

### 3.2. Preparation of PABA-Modified MWCNTs/Epoxy Resin Nanocomposites Via Photoinduced Frontal Polymerization

**Scheme 2:**
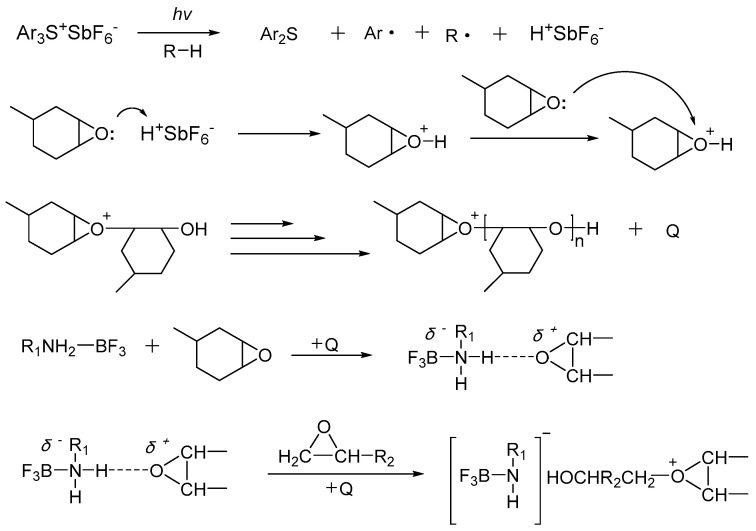
Mechanism of PIFP used for preparation of MWCNTs/epoxy nanocomposites.

To prepare the MWCNTs/epoxy nanocomposite using the PIFP method, 221-type alicyclic epoxy resin with a high heat-release ability was used as the resin matrix, mixed triarylhexafluoroantimonium salt was used as cationic photoinitiator, and boron trifluoride-amine complex was used as the TI. During the process, two types of reaction occurred successively. First a photolysis reaction occurred, during which an active strong acid was generated through the decomposition of photoinitiators under UV irradiation, and the epoxy groups in surface resin began a cationic ring-opening polymerization under the action of the strong acid. Second, a thermal curing of the epoxy resin occurred, during which the thermal initiator (boron trifluoride amine complex) decomposed rapidly due to the heat released in the first stage. The epoxy oligomers reacted with the BF3-amine to form an oxonium cation, and the ring-opening polymerization occurred in a chain mode according to the cationic reaction process, until the whole resin cured completely. The mechanism is shown in Scheme 2.

Front velocity (*V*_f_) and maximum temperature (*T*_max_) are two key parameters to be considered during a frontal polymerization reaction [41,42,43]. Suitable values of *V*_f_ and *T*_max_ must be achieved in order to realize a stable FP reaction.

**Figure 5:**
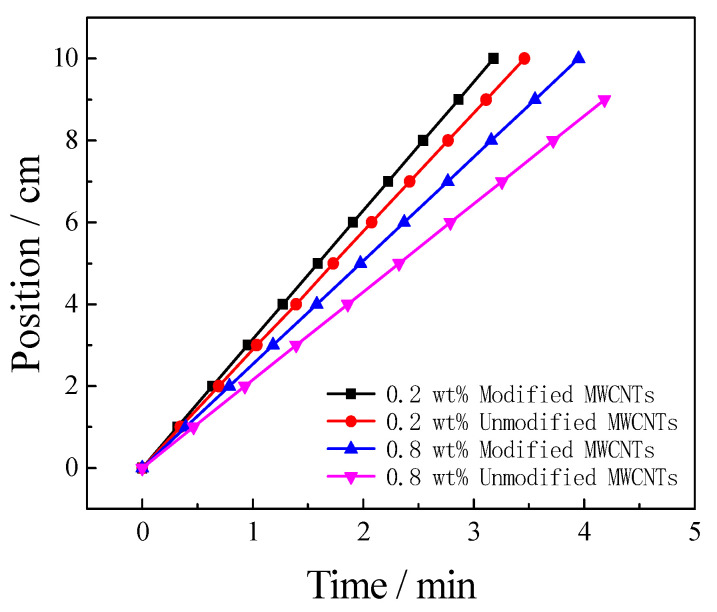
Front position versus time for the photoinduced FP of MWCNTs/epoxy resin.

Figure 5 plots the front position as a function of time during the preparation of MWCNTs/epoxy nanocomposites via photoinduced FP. The experimental data in all four sets conformed to well-fitted straight lines on the graph, indicating that the polymerization front propagated at a constant velocity, which is a typical feature of FP [34]. Table 1 lists the corresponding *V*_f_ values calculated according to the line slope. The data suggest a decreasing trend of *V*_f_ with the increase in the content of the fillers. One main reason could be the increase in viscosity of the mixture solution (as shown in Table 2). The higher the filler content, the greater the viscosity of the mixture system [44]. High viscosity hinders thermal diffusion, thus reducing the frontal velocity *V*_f_. The effect of conductive particles should also be considered [45,46]. When CNTs were added to the polymer, nanoparticles absorbed some amount of the released heat from the material (due to specific heat) and reduced the energy density, since less resin is available per unit volume. However, at the same time, the thermally conductive nanoparticles conducted the heat away from the front preheated the resin and accelerated the front reaction. These two phenomena had opposite effects on the frontal reaction. Since the front velocity of neat resin (0.81 cm/min) is much lower than that with MWCNTs added, it is thought that the addition of a small amount of conductive fillers is favorable in the frontal polymerization reaction. Additionally, the data in Table 1 show that the *V*_f_ of the PABA-modified MWCNTs/epoxy resin system was greater than that of the unmodified MWCNTs/epoxy resin system, although the viscosity of the unmodified MWCNTs/epoxy system was lower than that of modified MWCNTs/epoxy system. Except for the enhanced dispersibility and compatibility, the incorporation of carboxyl groups onto the surfaces of MWCNTs may play a crucial role in the frontal polymerization reaction, due to the change in exothermicity during the curing process caused by the reaction between the carboxyl groups and epoxy oligomers.

**Figure 6:**
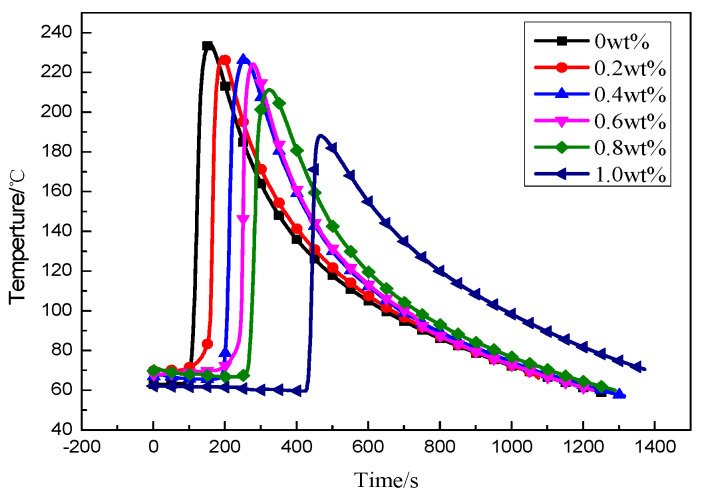
The temporal distribution of temperature of PABA-modified MWCNTs/epoxy reaction system with different modified MWCNTs contents.

Figure 6 shows the temporal distribution of temperature of the photoinduced FP of PABA-modified MWCNTs/epoxy resin system, and Table 3 shows the maximum temperature determined from the corresponding profile. The data show that the *T*_max_ value decreased with the increase in the content of the modified MWCNTs, which agrees well with the trend in frontal velocity, confirming a rule that a higher frontal reaction temperature *T*_max_ is needed to maintain the fast front reaction rate *V*_f_. Additionally, Figure S3 shows the *T*_max_ of the system with PABA-modified MWCNTs added is higher than that of the system with unmodified MWCNTs added, and the time needed to reach the highest reaction temperature *T*_max_ for the former is less than that needed for the latter, suggesting that the active groups on the surface of the modified MWCNTs participate in the FP reaction and help release more heat during the curing process. Therefore, we concluded the epoxy oligomers with modified MWCNTs added are better for heat conduction due to their enhanced dispersity and compatibility.

**Figure 7:**
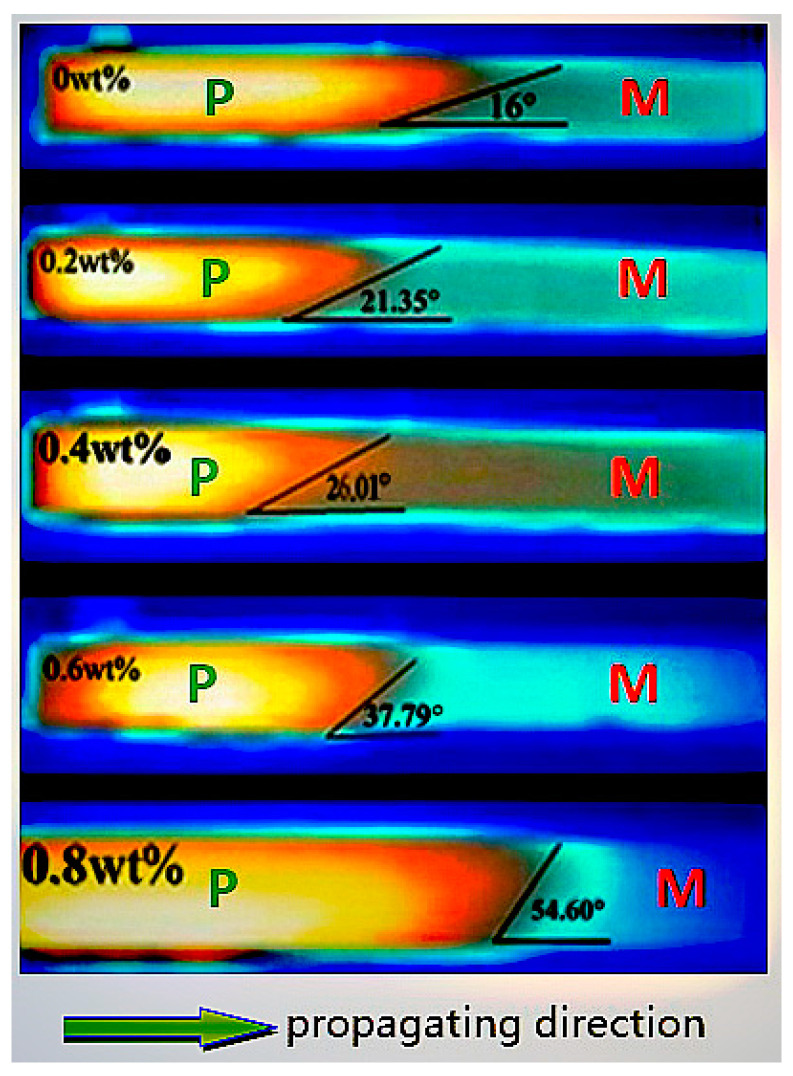
The propagating angles observed during the preparation of the epoxy resin nanocomposites via the PIFP approach with various contents of PABA-modified MWCNTs. These images were recorded using an infrared thermal imager. P, polymers (cured products); M, monomers (mixture of epoxy oligomers, PI, and TI). Each PIFP reaction propagated horizontally to the right.

A phenomenon known as propagating angle, which increases with the increase in addition of fillers, was previously discovered in frontal polymerization reactions [34]. In our study, such a feature was also observed. In our parallel experiments, a propagating angle of 16°, 21.35°, 26.01°, 37.79°, and 54.60° was clearly observed when the content of modified MWCNTs was 0, 0.2, 0.4, 0.6, and 0.8 wt %, respectively (Figure 7). The increase in the propagating angle and an increase in the filler loading was ascribed to the augment of the viscosity of the reaction system, which greatly inhibited the occurrence of thermal convection and reduced the *V*_f_ value. A slow propagating velocity usually accompanied a large propagating angle, resulting in uniform material properties.

### 3.3. Properties of Modified MWCNTs/Epoxy Resin Nanocomposite Prepared by Photoinduced Frontal Polymerization

**Figure 8:**
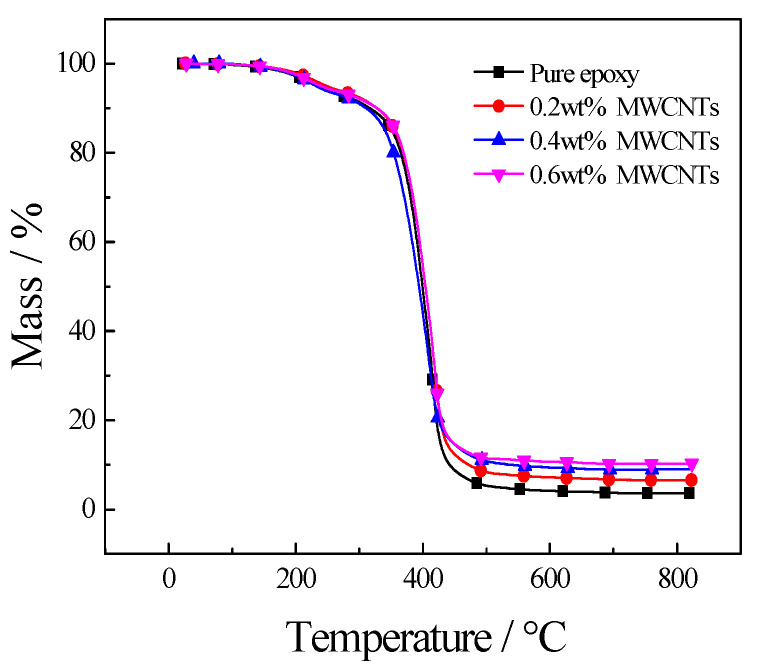
TGA curves of epoxy nanocomposites with the content of various PABA-modified MWCNTs.

Figure 8 shows the TGA curves of pure epoxy resin and its nanocomposites filled with various amounts of PABA-modified MWCNTs. The weight-loss rate of epoxy nanocomposites decreased as the content of modified MWCNTs increased. The condensate of pure epoxy resin substantially decomposed at ~520 °C, while the residual mass of the epoxy nanocomposites continued to increase when the loading of the modified MWCNTs increased. The residual mass of the three composites was 7.5%, 10.8%, and 12%, respectively, suggesting that the addition of modified MWCNTs enhanced the retention of the epoxy resin. Since MWCNTs resist heat and conduct temperatures well, the modified MWCNTs dispersed in the epoxy matrix absorbed and transferred energy to the external environment quickly once heated, resulting in better heat resistance in the corresponding nanocomposites.

**Figure 9:**
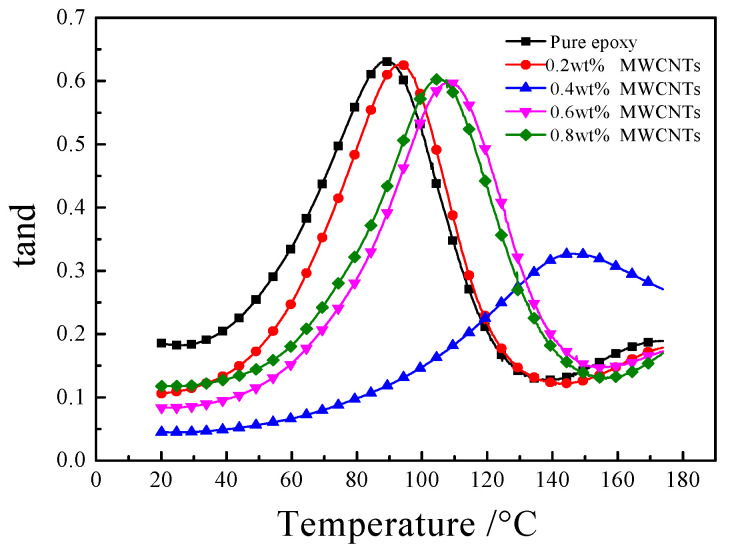
tanδ–*T* curves of epoxy nanocomposites with the content of various PABA-modified MWCNTs.

**Figure 10:**
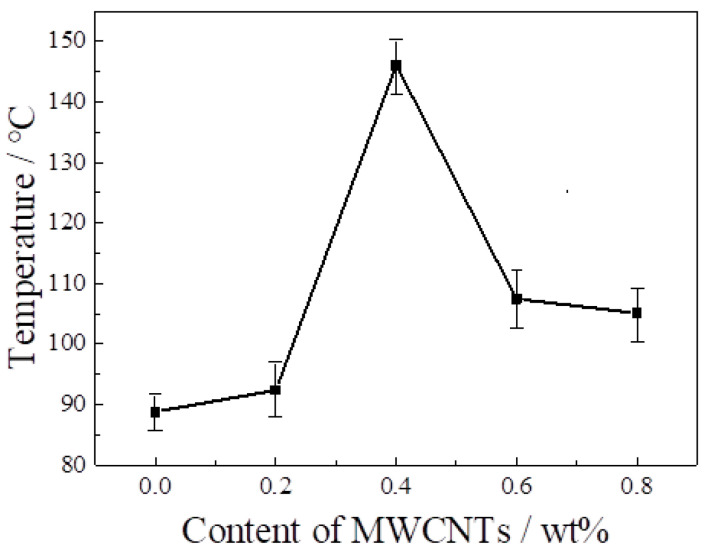
The *T_g_* of epoxy nanocomposites with the content of various PABA-modified MWCNTs.

Figure 9 shows the tanδ–*T* curves that were recorded in the DMA testing of the epoxy nanocomposites, and Figure 10 plots the corresponding glass transition temperature determined from the tanδ ~ *T* curves. The *T*_g_ value increased at first, but then decreased along with the increase in the MWCNTs content. The *T*_g_ of pure epoxy resin was 88.8 °C, but the *T*_g_ of the nanocomposites reached a maximum value of 146.1 °C when the content of modified MWCNTs reached 0.4 wt %. Even when the filler loading reached 0.8 wt %, the related *T*_g_ (105.1 °C) was still higher than for pure epoxy resin. These results indicate that the addition of modified MWCNTs can effectively enhance the heat resistance of the epoxy matrix, due to the strengthened interface between the matrix and the filler after the surface-carboxylation modification reaction.

**Figure 11:**
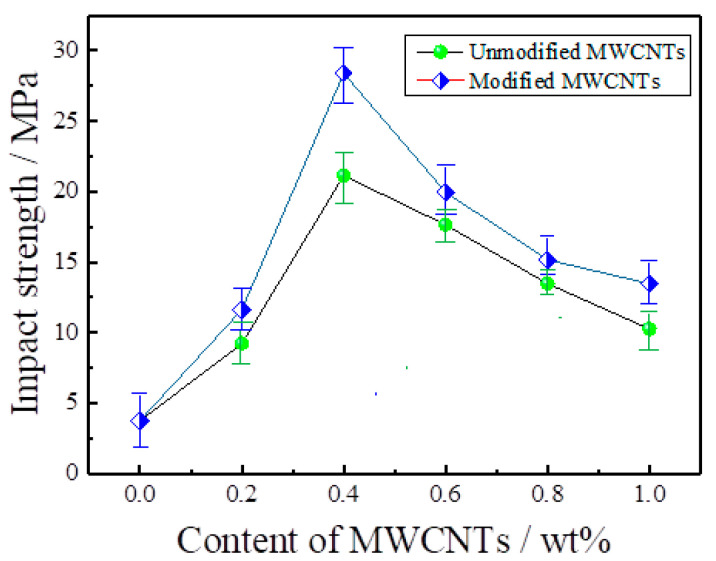
The impact strength of epoxy composites with the content of various PABA-modified MWCNTs.

The impact properties of the epoxy nanocomposites with various amount of PABA-modified MWCNTs added were tested (Figure 11). In comparison, the impact properties of the composites with various amounts of unmodified MWCNTs addition are also presented. The impact strength of the composites increased at first, and then decreased along with the increase in the loading of MWCNTs. When the content of the modified MWCNTs was 0.4 wt %, the impact strength jumped to its highest value (28.39 MPa), an increase of 462.23% compared to that of the pure epoxy resin (3.76 MPa), and 34.23% higher than that of the composite with 0.4 wt % unmodified MWCNTs added (21.14 MPa). The results showed that PABA-modified MWCNTs can effectively enhance the impact property of the epoxy composite better than unmodified MWCNTs. However, when the content of MWCNTs exceeded 0.4 wt %, the impact strength value of the composites began to decrease. This could be ascribed to the increase in the viscosity of the mixture, which reduced the dispersibility of the fillers [41].

**Figure 12:**
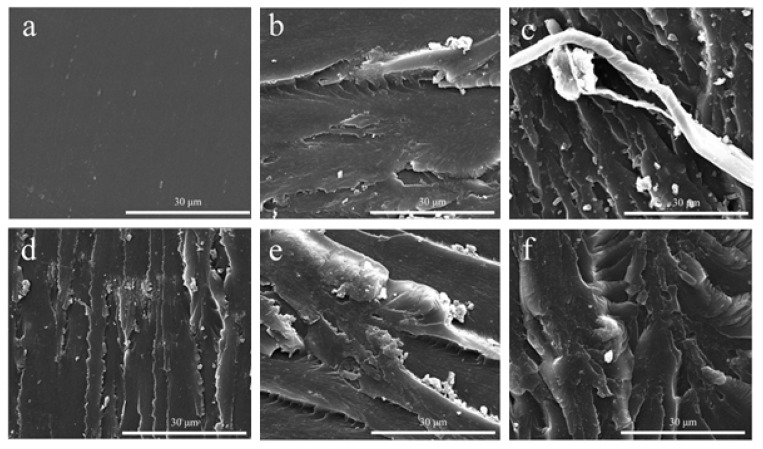
SEM images of the fracture of pure epoxy and its nanocomposite filled by PABA-modified MWCNTs: (**a**) pure epoxy resin; and (**b**) 0.2, (**c**) 0.4, (**d**) 0.6, (**e**) 0.8, and (**f**) 1.0 wt %.

Figure 12 shows an SEM photograph of the impact fracture of the epoxy nanocomposites with the content of various modified MWCNTs loaded. Smooth and flat features were observed in the sample of pure epoxy resin condensate, indicating a typical brittle fracture (Figure 12a). With modified MWCNTs added, the fracture of the nanocomposite samples became rougher, and displayed numerous scaly structures. This ductile characteristic resulted from the good compatibility, uniform dispersion, and enhanced interface bonding strength between the matrix and the fillers. When external force was applied, the carbon nanotubes developed a large number of microcracks. The network structure formed by the carbon nanotubes in the matrix resin effectively hindered crack propagation and absorbed the impact energy, thus enhancing the impact strength of the composite material. However, the agglomeration of fillers tended to occur when adding too many MWCNTs, leading to a drop in impact strength.

## 4. Conclusions

In this study, MWCNTs/epoxy resin nanocomposites were rapidly prepared using UV photoinduced frontal polymerization. After modification by PABA via carboxylation reaction, MWCNTs exhibited enhanced dispersibility and compatibility with the epoxy matrix. The addition of 0.4 wt % modified MWCNTs to epoxy oligomers greatly enhanced the toughness and heat resistance of the obtained nanocomposites, which demonstrated an increase of 462.23% in impact strength and an increase of 57.3 °C in the glass transition temperature *T*_g_ compared to pure epoxy resin condensate. The findings indicate that PIFP is a facile, rapid, and efficient approach for preparation of high-performance thermosetting nanocomposites, and it exhibits potential in the fields of fast repairing and deep curing.

## Figures and Tables

**Table 1:** The *V_f_* of the photoinduced FP of MWCNTs/epoxy resin.

Content of MWCNTs (wt %)	0	0.2	0.4	0.6	0.8	1.0
*V_f_* of Modified MWCNTs/Epoxy (cm/min)	0.81	3.15	3.04	2.72	2.54	1.43
*V_f_* of Unmodified MWCNTs/Epoxy (cm/min)	0.81	2.89	2.63	2.41	2.15	0.98

**Table 2:** Viscosity of the MWCNTs/epoxy resin mixture solution.

Content of MWCNTs (wt %)	0	0.2	0.4	0.6	0.8	1.0
Viscosity of Modified MWCNTs/Epoxy (mPa·s)	165	179	185	198	206	224
Viscosity of Unmodified MWCNTs/Epoxy (mPa·s)	165	133	139	146	165	192

**Table 3:** *T*_max_ of PABA-modified MWCNTs/epoxy resin mixture.

Content of Modified MWCNTs (wt %)	0%	0.2%	0.4%	0.6%	0.8%
*T*_max_ (°C)	234.5	227.9	226.6	224.2	211.3

## Supplementary Materials

The following are available online at https://www.mdpi.com/1996-1944/13/24/5838/s1, Figure S1: Scheme of the device used for photoinduced frontal polymerization; Figure S2: TGA curves of pure epoxy resin; Figure S3: The temporal distribution of temperature of the unmodified MWCNTs/epoxy and PABA-modified MWCNTs/epoxy reaction system with different filler loading.
