# Supplementary material for: Rapid Preparation of MWCNTs/Epoxy Resin Nanocomposites by Photoinduced Frontal Polymerization
Source: Materials (Basel). 2020 Dec 21;13(24):5838. doi: 10.3390/ma13245838 (PMC7767450; doi:10.3390/ma13245838)
Supplement: Supplementary file 1 [file materials-13-05838-s001.pdf]

materials

Supplementary Materials: Rapid Preparation of MWCNTs/Epoxy Resin Nanocomposites by Photoinduced Frontal Polymerization

Guofeng Hu [1], Wanli Fu [1,2], Yumin Ma [1], Jianping Zhou [1,3,*], Hongbo Liang [1,3], Xinmei Kang [4] and Xiaolin Qi [4]

[1] School of Material Science and Engineering, Nanchang Hangkong University, Nanchang 330063, China; 1801085204012@stu.nchu.edu.cn (G.H.); 70965@nchu.edu.cn (W.F.); maym@hollowlite.com (Y.M.); lhongbo@nchu.edu.cn (H.L.)
[2] State-owned Assets Management Division, Nanchang Hangkong University, Nanchang 330063, China;
[3] Jiangxi Provincial Engineering Research Center for Surface Technology of Aeronautical Materials, Nanchang Hangkong University, Nanchang 330063, China
[4] Aviation Key Laboratory of Science and Technology on Life-support Technology, Xiangyang 441000, China; kangkang198306@163.com (X.K.); ajian421@126.com (X.Q.)
* Correspondence: zhoujp@nchu.edu.cn

Received: 25 October 2020; Accepted: 15 December 2020; Published: 21 December 2020

Rapid Preparation of MWCNTs/Epoxy Resin Nanocomposites by Photoinduced Frontal Polymerization

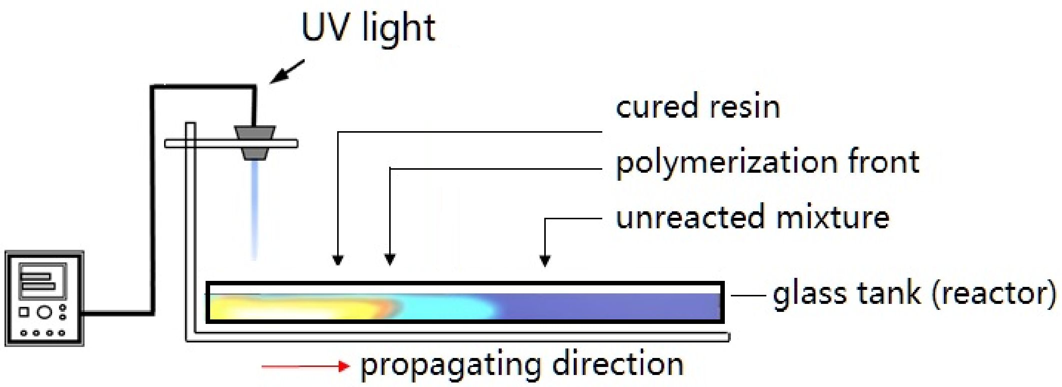

Figure S1. Scheme of the device used for photoinduced frontal polymerization.

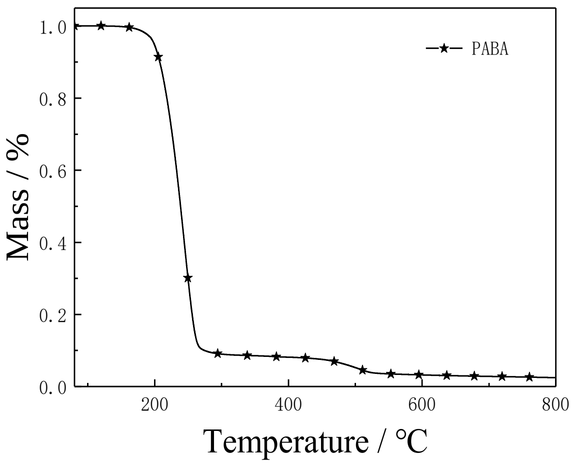

Figure S2. TGA curves of pure epoxy resin.

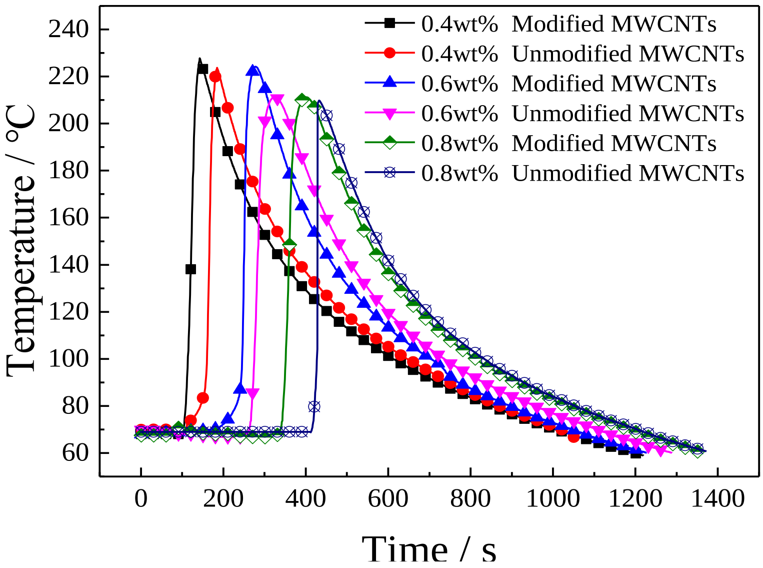

Figure S3. The temporal distribution of temperature of the unmodified MWCNTs/epoxy and PABA-modified MWCNTs/epoxy reaction system with different filler loading.

Publisher's Note: MDPI stays neutral with regard to jurisdictional claims in published maps and institutional affiliations.

© 2020 by the authors. Licensee MDPI, Basel, Switzerland. This article is an open access article distributed under the terms and conditions of the Creative Commons Attribution (CC BY) license (http://creativecommons.org/licenses/by/4.0/).
